# Intravenous immunoglobulin therapy could have efficacy in severe sepsis

**DOI:** 10.1186/cc12939

**Published:** 2013-11-05

**Authors:** Ahmin Kim, Mari Yokota, Noriko Saito, Munekazu Takeda, Tomoyuki Harada, Mizuho Namiki, Arino Yaguchi

**Affiliations:** 1 Department of Critical Care and Emergency Medicine, Tokyo Women's Medical University, Tokyo, Japan

## Background

Intravenous immunoglobulin (IVIG) administration has been approved to use for severe sepsis with antibiotics by the Ministry of Health, Labour and Welfare since 1980 in Japan. IVIGs are commonly used for severe sepsis and septic shock in Japan, while the international guidelines for management of severe sepsis and septic shock in 2012 suggest not using IVIG in adult patients. Our hypothesis is that IVIG administration has an efficacy for severe sepsis and septic shock.

## Materials and methods

This retrospective observational study included all adult patients in our ICU who were administered IVIG for severe sepsis and septic shock from January 2011 to June 2013. IVIG was used at 5,000 mg/day every 24 hours for 3 days. We compared body temperature (°C), WBC (/mm^3^), CRP (mg/dl), procalcitonin (ng/ml) and serum immunoglobulin G (IgG) (mg/dl; normal >870) between before and after IVIG treatment. Values are expressed as the median. The Wilcoxon signed-rank test was used for the statistical analysis. *P *< 0.05 was considered significant.

## Results

One hundred and fifty-one patients (85 men, 66 women; age range 23 to 96 (median 67.8)) were included in this study. The 28-day mortality after IVIG treatment was 13.9%. The SOFA score before IVIG treatment was 5.0. Values of WBC, CRP and procalcitonin were significantly decreased after IVIG treatment (10,905 vs. 9,805, *P *< 0.0001, 12.3 vs. 7.7, *P *< 0.0001, 2.4 vs. 1.7, *P *= 0.0003, respectively). Body temperature did not significantly change (37.4 vs. 37.2, *P *= 0.07). Serum IgG was significantly increased after the treatment (1,046 vs. 1,563, *P *= 0.003).

## Conclusions

The present study has some limitations because of being a retrospective observational study. However, the mortality was quite low in the group of patients included in this study. Moreover, after IVIG treatment values of WBC, CRP and procalcitonin were improved. The median value of serum IgG before treatment was within the normal range, but after treatment was also significantly improved. There is a possibility that severe septic patients require additional IgG regardless of its normal concentrations in their blood.

